# Meristems clarify fuzzy floral boundaries: a commentary on ‘Are capitula inflorescences? A reassessment based on flower-like meristem identity and ray flower development’

**DOI:** 10.1093/aob/mcaf256

**Published:** 2025-10-16

**Authors:** Paula J Rudall, Richard M Bateman

**Affiliations:** Jodrell Laboratory, Royal Botanic Gardens, Kew, Richmond, Surrey TW9 3AB, UK; Jodrell Laboratory, Royal Botanic Gardens, Kew, Richmond, Surrey TW9 3AB, UK

Some of the best botanical science emerges from studies that address a specific question through observations of a particular well-chosen study group but that ultimately yield results with implications that extend well beyond the original hypothesis. Such is the case with the paper published in this issue of *Annals of Botany* by Claßen-Bockhoff *et al*. (2025), entitled ‘Are capitula inflorescences?’, where the authors employ comparative ontogenetic data to document the developmental trajectories of several representative members of the daisy family (Asteraceae). When placed in a developmental–genetic context, the data allow the authors to explore the possible evolutionary origins of the daisy’s characteristic flower-like capitulum, which is composed of numerous, tightly packed flowers that are typically dimorphic: petaloid bilaterally symmetrical ‘ray flowers’ surround non-petaloid radially symmetrical ‘disc-flowers’.

To the eyes of many observers, flowers and inflorescences are strictly non-homologous structures. Capitula are widely regarded as classic examples of pseudanthia, which traditionally have been defined as structures that resemble flowers but are actually judged homologous to inflorescences. However, the pivotal insight achieved by Claßen-Bockhoff and co-workers lies in recognizing the crucial presence and role of a novel category of reproductive meristem, the floral unit meristem (FUM *sensu* Claßen-Bockhoff and Bull-Hereñu, 2013), which effectively expands the evolutionary potential of reproductive developmental processes. Claßen-Bockhoff *et al*. (2025) elaborate their concept of three types of reproductive meristem, one of them indeterminate (IMs), the other two determinate (FUMs and flower meristems: FMs). They infer that the daisy capitulum develops from an FUM, which itself evolved through a single developmental shift from an indeterminate to a determinate meristem. Crucially, in their interpretation, this solitary shift replaces the several incremental steps required by traditional hypotheses of pseudanthial origin, as well as necessitating a redefinition of pseudanthia.

**Fig. 1.**
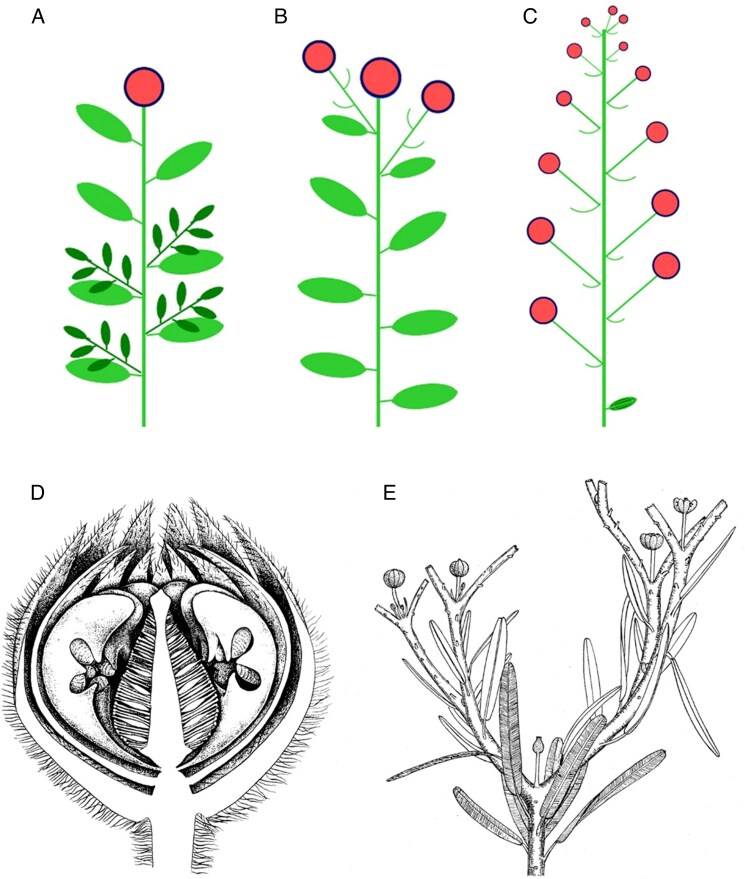
(A–C) Comparison of the basic architectures of inflorescences, showing flowers as red circles: (A) solitary terminal flower on a leafy shoot; (B) cymose branching, showing a terminal flower with lateral branches subtended by bracts; (C) simple racemose inflorescence in which flowers open acropetally; a terminal flower never develops and the meristem eventually becomes exhausted. (D, E) Diagrams of anatomically preserved reproductive structures in the Jurassic bennettitalean gymnosperm *Williamsoniella coronata*: (D) section of a ‘flower’ (source: Crane, 1985); (E) part of an ‘inflorescence’, with some leaves removed (source: Thomas, 1916).

Classical morphology distinguishes between determinate (‘closed’) and indeterminate (‘open’) inflorescences (Weberling, 1992). In a determinate inflorescence such as a solitary terminal flower (Fig. 1A) or a cyme (Fig. 1B), all of the axes terminate in a flower. In contrast, in an indeterminate inflorescence such as a raceme (Fig. 1C), the meristem forms nodes and internodes by acropetal extension growth; it generates flowers by lateral segregation until it becomes exhausted, never forming a terminal flower. These two developmental trajectories encompass a strikingly diverse spectrum of inflorescence architectures, including panicles and thyrses. In addition to external morphology, the internal cellular arrangements of the apical zones also differ. Those of indeterminate meristems resemble vegetative meristems, focused on a ‘central mother-cell zone’ of self-renewing stem cells that confer indeterminacy. Determinate meristems (including FMs and FUMs) lack a pluripotent central zone and are determinate from inception. Determinate inflorescence meristems develop by expansion and fractionation; both FUMs and FMs possess a flattened or conical receptacle. FUMs resemble FMs except that they can fractionate into secondary FUMs or FMs. Thus, in this concept, daisy capitula equate with FUMs, raising the argument that FUMs could legitimately be equated with pseudanthia *per se*.

Can the FUM concept be extended to examine the origin and early evolution of inflorescences and flowers in angiosperms? This question is far less simple than it sounds, as is evident from the complex traditional terminology accumulated by botanists in their attempts to accurately describe inflorescences and to clarify their boundaries with true flowers. Evolutionary–developmental genetics has in recent years given us a much deeper understanding of the MADS-box genes that determine the fate and spatial organization of floral organ primordia, epitomized by the pivotal ‘ABC’ model and its derivatives, in which the C-class (*AGAMOUS*-like) genes not only interact with B-class genes to determine carpel identity but also ensure the determinacy of the floral axis (e.g. Coen and Meyerowitz, 1991). In contrast, the wide spectrum of inflorescence architectures has proved far less tractable to modelling. The key genes that regulate inflorescences are *TERMINAL FLOWER1* (*TFL1*), *LEAFY* (*LFY*) and *APETALA1* (*AP1*); differences in the form of closed inflorescences can be explained by evolutionary shifts in the respective expression levels of *TFL1*-like and *AP1*-like genes, which interact with other genes to specify FM identity via a series of feedback loops (Ma *et al*., 2017).

Although the phylogenetic context among angiosperms has changed through recent decades, it is likely that the determinate inflorescence – perhaps a solitary terminal flower – represents the ancestral type, though many transitions have occurred within numerous clades (Rudall and Bateman, 2010). Indeed, FUMs are apparently widespread across the angiosperm phylogeny. For example, Claßen-Bockhoff and co-workers have documented FUMs in detail in several eudicot families in addition to Asteraceae (Claßen-Bockhoff and Bull-Hereñu, 2013; Baczyński *et al*., 2022). They supplemented their observations with data from other sources, not only for monocots and magnoliids, but also for some of the ANA-grade families that consistently constitute the earliest-diverging lineages in molecular phylogenies.

For example, in *Amborella*, which is placed as the sister taxon to all other angiosperms in many molecular analyses, the inflorescence is determinate and resembles an FUM; the young flower or inflorescence axis is initially conical but expands laterally to form a flat floral meristem with marginal initiation of appendages (Posluszny and Tomlinson, 2003). Furthermore, reproductive meristems in the ANA-grade family Hydatellaceae (Nymphaeales) cannot readily be interpreted as either flowers or cymose inflorescences, despite using criteria ranging from comparative morphology to gene expression, since LFY proteins are localized in reproductive primordia at several different hierarchical levels (Rudall *et al*., 2009). In their influential attempt to reconstruct phylogenetically the morphology of the ancestral angiosperm flower, Sauquet *et al*. (2017) treated pseudanthia in general as inflorescences rather than flowers and chose to score Hydatellaceae as non-interpretable. Rudall *et al*. (2009) described these ambiguous structures as ‘non-flowers’, perhaps resembling a prefloral condition; they hypothesized iterative hierarchical shifts between flowers and inflorescences, a version of developmental heterochrony that is directly relatable to the FUM concept. Indeed, the highly iterative nature of this transition implies that the FUM was a major evolutionary innovation.

We might then move down the seed-plant phylogeny to consider the probable conditions in candidate groups still competing to be perceived as the closest relatives of angiosperms. Most molecular phylogenies based exclusively on extant taxa are unhelpful, placing angiosperms as sister to all other extant groups of seed-plants (e.g. Magallon *et al*., 2013). However, comparable but contrasting morphological phylogenies (e.g. Hilton and Bateman, 2006) have persistently placed some intriguing extinct groups known only from fossils, such as Bennettitales and Caytoniales, as potential angiosperm-sisters. In particular, many Bennettitales possessed terminal flower-like structures that were either solitary or arranged in cymose clusters (Rudall and Bateman, 2010). These reproductive structures can be readily envisaged as having developed via a laterally extensive FUM-like meristem, generating the conical/discoid receptacles of Mesozoic bennettites such as *Cycadeoidea* and *Williamsoniella* (Fig. 1D, E: structurally a genuine flower by some definitions: Bateman *et al*., 2006).

Viewed from a more process-based perspective, the developmental model envisaged for the Asteraceae capitulum is appealing because it confers important yet complementary roles on heterochrony, spatial/geometric constraints and auxin clines. Clines in auxin across the capitulum prompt competition among peripheral bracts, ray florets and disc florets that in extreme cases can suppress one of these organ categories (Zhang *et al*., 2021). Similarly for Fibonacci spirals, the popular idea that the near-perfect packing of the disc flowers within sunflower pseudanthia is pre-programmed gives way to a more pragmatic hypothesis based not on mathematical perfection but rather on transient local auxin maxima generating crowded flower primordia (FMs) that are forced to jostle for position until the underpinning FUM is exhausted. In other words, the impressively precise floral patterning of Asteraceae is the emergent product of spatial constraint acting upon meristematic behaviour that in turn reflects transient auxin profiles. Thus, the morphological end-point is separated from any underlying change(s) in gene expression by a crucial series of epigenetic filters.

Such developmental systems are vulnerable to threshold effects that could reflect even subtle changes in developmental context, leading to one or more heterochronic shifts in the timing of transitions from indeterminacy to determinacy. Assuming heritability, such shifts would have the potential to alter any or all aspects of development downstream of the initial change (i.e. could cause extensive pleiotropy and so greatly modify the resultant phenotype). Such auxin-mediated transformations could potentially emerge across a single generation and, at least initially, without the need for strong directional or disruptive selection (Bateman and DiMichele, 2002). A high diversity of inflorescences would easily and simply evolve, helping to explain the frequency of evolutionary transitions to pseudanthia implied by the molecular phylogenies of angiosperms (e.g. Baczyński and Claßen-Bockhoff, 2023, fig. 3).

Classical terminology can sometimes help to describe morphological diversity, but it can also merely end-run rather than resolve questions of inflorescence evolution. The multiplicity of meristem categories involved in inflorescence ontogeny together constitute a less overt developmental system than that governing simple *bona fide* flowers – one where architecture, shape and size interact to allow a greater role for epigenetic processes, limiting some evolutionary possibilities while simultaneously opening the door to others. Developmental morphologists raised on the ABC model of flower development, formulated under the influence of only a single category of meristem (FM), are now successfully grasping the nettle of inflorescence ontogeny (e.g. Ma *et al*., 2017). Nonetheless, they may be obliged by the FUM hypothesis to widen their horizons still further, with regard to both homology assessment and modes of gene expression.
